# From biomarker discovery to prediction models: clinical translation of multi-omics for early prediction of gestational diabetes mellitus

**DOI:** 10.3389/fendo.2026.2007266

**Published:** 2026-10-22

**Authors:** Huibo Yan

**Affiliations:** Clinical Laboratory, Shanghai Putuo District Maternity and Infant Hospital, Shanghai, China

**Keywords:** biomarker, early prediction, first trimester, gestational diabetes mellitus, gut microbiome, lipidomics, metabolomics, prediction model

## Abstract

Gestational diabetes mellitus (GDM) affects roughly one in seven pregnancies worldwide, yet diagnosis by oral glucose tolerance testing at 24–28 weeks of gestation leaves only a narrow window for intervention. The demonstrated benefit of treating early-diagnosed GDM has intensified interest in first-trimester prediction. This narrative review synthesises recent advances in early-pregnancy biomarkers for GDM, shifting the emphasis from biomarker discovery to clinical translation. Conventional lipid indicators and composite indices provide moderate predictive power at minimal cost (odds ratios 1.4–1.7; AUCs 0.75–0.82). Metabolomic and lipidomic studies have identified recurrent signatures — phospholipid remodelling, glycerolipid accumulation, fatty acid and acylcarnitine excess, branched-chain amino acids and bile acids — that map onto insulin resistance, lipotoxicity and hepatic steatosis, and omics-enhanced models report derivation-stage AUCs of approximately 0.83–0.95 with apparent incremental value over clinical factors alone, although these estimates derive predominantly from small cohorts that have rarely undergone external validation. Gut-microbiome features, thyroid parameters and environmental exposures further widen the aetiological frame. Translation remains limited, however, by small derivation cohorts, scarce external validation, analytical heterogeneity and the absence of trials testing biomarker-guided early intervention. We propose a pragmatic roadmap towards clinically deployable first-trimester prediction, with particular attention to high-burden Asian populations.

## 1 Introduction

Gestational diabetes mellitus (GDM), defined as hyperglycaemia first detected during pregnancy that does not meet the diagnostic thresholds for overt diabetes, is one of the most common medical complications of pregnancy (1). Its prevalence has risen steadily over recent decades in parallel with the global epidemics of obesity and type 2 diabetes, and GDM is now estimated to affect roughly one in seven pregnancies worldwide, although reported rates vary considerably across populations and diagnostic criteria (2, 3). In mainland China, a meta-analysis applying the International Association of Diabetes and Pregnancy Study Groups (IADPSG) criteria estimated the pooled prevalence at 14.8% (95% CI 12.8–16.7%) (4).

The clinical importance of GDM extends well beyond pregnancy itself. The landmark Hyperglycemia and Adverse Pregnancy Outcomes (HAPO) study demonstrated continuous, graded associations between maternal glycaemia and adverse outcomes — including large-for-gestational-age birth, caesarean delivery, neonatal hypoglycaemia and pre-eclampsia — with no obvious inflection points (5). In the long term, women with a history of GDM face an approximately seven-fold higher risk of developing type 2 diabetes (6, 7) and an elevated risk of cardiovascular and cerebrovascular disease (8), while their offspring are more likely to develop childhood adiposity and impaired glucose metabolism by early adolescence (9).

Despite this burden, the diagnosis of GDM still relies on a 75-g oral glucose tolerance test (OGTT) performed at 24–28 weeks of gestation, as recommended by the IADPSG, the World Health Organization (WHO) and the American Diabetes Association (ADA) (1, 10, 11). Although randomised trials have confirmed that treatment initiated at this stage improves perinatal outcomes (12, 13), diagnosis in the late second trimester leaves a narrow window for intervention, and accumulating evidence indicates that metabolic dysregulation precedes the clinical diagnosis by weeks to months (14). The Treatment of Booking GDM (TOBOGM) trial recently showed that immediate treatment of GDM diagnosed before 20 weeks’ gestation modestly reduced a composite of adverse neonatal outcomes compared with deferred or no treatment (24.9% vs 30.5%; adjusted risk difference −5.6 percentage points) (15), providing direct randomised evidence in favour of earlier identification.

These developments have catalysed intense interest in first-trimester prediction of GDM. Beyond traditional risk factors and routine lipid indicators, the past two years have seen a rapid accumulation of studies applying metabolomics, lipidomics and gut-microbiome profiling to maternal blood in early pregnancy (16–18). A recent scoping review catalogued 145 candidate first-trimester biomarkers across 133 studies, yet also exposed substantial heterogeneity in analytical platforms, gestational windows and reporting standards (19). Translation of these candidates into clinically usable prediction models remains limited: most biomarkers have not been externally validated, and few models have progressed beyond derivation cohorts.

In this narrative review, we shift the emphasis from biomarker discovery to clinical translation. We synthesise recent evidence on conventional lipid indicators and composite indices, free fatty acid profiles, metabolomic and lipidomic signatures, and gut-microbiome-derived metabolites for the early prediction of GDM, with particular attention to emerging data from Asian populations, in whom insulin resistance and type 2 diabetes risk emerge earlier and at lower body mass index (16, 17). We further compare the performance of prediction models built on clinical variables alone, omics signatures alone, and their combination, and discuss the analytical, statistical and logistical barriers that must be overcome before first-trimester prediction can be integrated into routine antenatal care.

Literature search strategy. This narrative review was informed by structured searches of PubMed/MEDLINE and Embase from database inception to 30 June 2026, combining the terms “gestational diabetes”, “GDM”, “first trimester”, “early pregnancy”, “prediction”, “prediction model”, “metabolom*”, “lipidom*”, “biomarker”, “microbiome” and “machine learning”. Priority was given to peer-reviewed original studies published in English that sampled women before 20 weeks of gestation and reported either prediction of incident GDM or quantitative associations with incident GDM; landmark trials and authoritative guidance (HAPO, TOBOGM, GEMS, and ADA/WHO/IADPSG statements) were included for clinical context. The reference lists of retrieved articles and of the 2025 scoping review by Swinburne et al. were screened for additional eligible studies. Because this is a narrative rather than a systematic review, no PRISMA flow diagram was constructed and the possibility of selection bias cannot be excluded. Quantitative results are therefore presented study-by-study in **Tables 1**–**3** rather than as pooled estimates, and exploratory associations are distinguished from validated clinical evidence throughout.

**Table 1:** Studies of conventional lipid indicators and composite indices in early pregnancy and GDM.

Study	Population/cohort	N analysed	Indicator(s)	Key results	Validation
Tang 2025 (20)	Changsha, China; prospective cohort	32,411	TG, TC, remnant cholesterol	Q4 vs Q1: OR 1.65/1.65/1.40; the same lipids also predicted pre-eclampsia	Not reported
Wang 2026 (21)	Guilin, China; case–control development (First Affiliated Hospital) plus independent external cohort (Second Affiliated Hospital); Mendelian randomisation	Development 1,274 (553 GDM/721 controls); external 390 (180/210)	Nomogram: BMI, TG, TC, LDL-c, HbA1c	AUC 0.772 (training), 0.754 (internal validation), 0.759 (external validation); calibration plots and decision-curve analysis reported	Random-split internal validation plus genuine external validation (n = 390)
Xu 2025 (22)	Korea (Seoul; secondary analysis of the prospective “Fatty Liver in Pregnancy” registry, NCT02276144)	585 (36 GDM); enrolled 663 → analytic 585	ZJU index (FPG, TG, ALT/AST, BMI)	AUC 0.802 (95% CI 0.72–0.88); Youden cut-off 33.64, sensitivity 0.722, specificity 0.758; also associated with NAFLD	None (sensitivity analyses only: propensity-score matching, Firth regression)
Zhao 2026 (23)	Kunshan, China; single-centre retrospective cohort of women with established GDM (TRIPOD-adherent)	587 (all GDM; 192 developed late-pregnancy HTG)	Pre-gravid BMI, FPG and 1hPG at GDM diagnosis, first-trimester TG and HDL-C	Outcome: late-pregnancy hypertriglyceridaemia (TG ≥2.3 mmol/L at 32–36 wk), NOT incident GDM; AUC 0.816 (95% CI 0.754–0.878) in held-out test set; calibration slope 0.94; Brier score 0.151; decision-curve analysis positive	7:3 split-sample test set plus 1000× bootstrap optimism correction (optimism-corrected AUC 0.805); no external validation

AUC, area under the receiver operating characteristic curve; BMI, body mass index; FPG, fasting plasma glucose; GDM, gestational diabetes mellitus; HTG, hypertriglyceridaemia; MR, Mendelian randomisation; NAFLD, non-alcoholic fatty liver disease; OGTT, oral glucose tolerance test; OR, odds ratio; Q, quartile; RC, remnant cholesterol; TC, total cholesterol; TG, triglycerides; ZJU, Zhejiang University.

**Table 2:** First-trimester metabolomic and lipidomic studies for early prediction of GDM.

Study	Cohort/design	N analysed (parent cohort)	Sampling window	Key findings	Model performance (AUC, 95% CI)
Masalin 2024 (24)	Finland, EDDIE population-based prospective cohort; nested case–control	210 GDM (126 E-GDM/84 L-GDM) vs 209 controls (parent n = 1,605)	Serum at 12–16 wk (early OGTT1 visit)	Long-chain fatty acids and medium/long-chain acylcarnitines correlated with glycaemia; HOMA-IR with tyrosine and phospholipids; eGDM vs l-GDM differences mainly in women with overweight; untargeted UHPLC-MS	No prospective prediction model for incident GDM; PLS-DA classification (existing subgroup vs matched controls): AUC 0.76 (normal-weight E-GDM), 0.73 (normal-weight L-GDM), 0.73 (overweight E-GDM), 0.68 (overweight L-GDM)
Tenenbaum-Gavish 2020 (25)	Israel; ASPRE-derived single-centre prospective dataset	20 GDM vs 185 controls (dataset 256 screened → 205 analysed)	11 + 0–13 + 6 wk	Two exploratory derivation models: with BMI (insulin, sCD163, TNF-α) intended for obese women; without BMI (sCD163, insulin, TNF-α, PP13, PAPP-A) intended for non-obese women	0.95 (0.88–0.99), DR 89% at 10% FPR (with BMI); 0.94 (0.85–0.99), DR 83% at 10% FPR (without BMI). Derivation only — no validation; authors explicitly recommend independent validation
Jung 2025 (17)	Korea, “Fatty Liver in Pregnancy” registry (1,936 enrolled → 1,756 eligible); nested case–control	118 vs 118	10–14 wk (second samples at 24–28 wk)	Altered conjugated bile acids, amino acids, PE/PI lipids; exploratory mediation via MASLD (PM 9.7–31.9%); multi-platform UPLC-MS	Clinical factors alone 0.631 (0.566–0.693) → clinical + metabolites 0.849 (0.797–0.892), DeLong p < 0.001; no external validation
Sharma 2025 (16)	India, STRiDE prospective cohort; nested case–control + machine learning	50 GDM vs 50 controls (parent n = 2,115)	< 16 wk	49 associated metabolites (phosphatidylcholines, sphingomyelins, triacylglycerols); random-forest 8-metabolite panel	ROC-analysis AUC 0.880 (0.809–0.951), sensitivity 82%, specificity 88%; bootstrap mean AUC 0.83 (0.77–0.89); single-split test estimates up to 0.98; clinical-only models 0.655 (0.547–0.763)/0.688 (0.582–0.794); integrated model 0.881 (0.810–0.952); no external validation
Saadati 2026 (18)	TOBOGM multicentre RCT; single-site (Monash, Australia) sub-study; cross-sectional	89 eGDM vs 91 non-GDM controls (sub-study n = 180)	Fasting plasma at diagnostic OGTT, 10–19 + 6 wk	543 lipids across 18 classes; concurrent eGDM signature: DAG/FA/PE/LPE ↑, LPC/ether-PC/HexCer ↓; untargeted LC-MS (Orbitrap)	No prediction model for incident GDM (concurrent molecular profiling at diagnosis)

DG, diacylglycerol; eGDM, early-onset gestational diabetes mellitus; FA, fatty acid; FPR, false-positive rate; HexCer, hexosylceramide; LC-MS, liquid chromatography–mass spectrometry; LPC, lysophosphatidylcholine; L-GDM, late-onset gestational diabetes mellitus; MASLD, metabolic dysfunction-associated steatotic liver disease; PAPP-A, pregnancy-associated plasma protein A; PC, phosphatidylcholine; PE, phosphatidylethanolamine; PM, proportion mediated; RCT, randomised controlled trial; sCD163, soluble cluster of differentiation 163; SM, sphingomyelin; TAG, triacylglycerol; TNF-α, tumour necrosis factor-α; (U)HPLC-MS(/MS), (ultra-) high-performance liquid chromatography–(tandem) mass spectrometry. Cohort acronyms (ASPRE, EDDIP, STRiDE, TOBOGM) are as designated in the original publications.

**Table 3:** Performance of GDM prediction models, stratified by input type.

Model class	Study	Predictors	AUC (95% CI)	Validation type
Clinical factors only	Jung 2025 (17)	Maternal clinical risk factors	0.631 (0.566–0.693)	Derivation (within-study comparison)
Clinical factors only	Sharma 2025 (Models 1a/1b) (16)	Age, BMI or waist-to-height ratio, family history, HbA1c	0.655 (0.547–0.763)/0.688 (0.582–0.794)	Derivation
Routine indices/composite	Wang 2026 (21)	BMI, TG, TC, LDL-c, HbA1c	0.772/0.754 (internal); 0.759 (external)	Random-split internal + external (n = 390)
Routine indices/composite	Xu 2025 (22)	ZJU index	0.802 (0.72–0.88)	None
Omics-enhanced	Jung 2025 (17)	Clinical factors + 16 selected metabolites	0.849 (0.797–0.892)	Derivation (within-study comparison)
Omics-enhanced	Sharma 2025 (16)	8-metabolite random-forest panel	0.880 (0.809–0.951); bootstrap mean 0.83 (0.77–0.89)	Iterated split-sample + bootstrap; no external validation
Omics + clinical (integrated)	Sharma 2025 (Model 1c) (16)	Conventional risk factors + 8-metabolite panel	0.881 (0.810–0.952)	Derivation
Omics-enhanced	Tenenbaum-Gavish 2020 (25)	BMI + insulin + sCD163 + TNF-α (obese); sCD163 + insulin + TNF-α + PP13 + PAPP-A (non-obese)	0.95 (0.88–0.99)/0.94 (0.85–0.99)	None — derivation only; 20 GDM cases; independent validation explicitly recommended by the authors

Where two AUC values are shown, the first refers to derivation (or training) performance and the second to validation performance. —, not reported or not applicable. AUC, area under the receiver operating characteristic curve; BMI, body mass index; GDM, gestational diabetes mellitus; HTG, hypertriglyceridaemia; OGTT, oral glucose tolerance test; ZJU, Zhejiang University.

## 2 Evolution of diagnostic criteria: why 24–28 weeks is late

The diagnostic criteria for GDM have been debated for more than half a century. Early criteria, derived from the work of O’Sullivan, were designed to predict future maternal diabetes rather than pregnancy outcomes, and multiple competing standards coexisted for decades (3). The HAPO study provided the first outcome-based evidence, showing that the risks of adverse pregnancy outcomes rise continuously across the full range of maternal glucose values (5). On this basis, the IADPSG in 2010 proposed a one-step 75-g OGTT with thresholds of 5.1 mmol/L (fasting), 10.0 mmol/L (1-h) and 8.5 mmol/L (2-h), with a single abnormal value sufficing for diagnosis (10); these thresholds were adopted by the WHO in 2013, which further distinguished GDM from diabetes in pregnancy (DIP) (1). The ADA accepts both the one-step and the two-step strategy (11). Yet the controversy is not settled: in the GEMS trial, randomisation to lower versus higher glycaemic criteria more than doubled the diagnosis rate (15.3% vs 6.1%) without reducing the incidence of large-for-gestational-age infants (adjusted relative risk 0.98), although women whose glucose values fell between the two criteria sets appeared to benefit from treatment (26).

The timing of the diagnostic OGTT — 24 to 28 weeks of gestation — has a physiological rationale. Early pregnancy is a period of enhanced insulin sensitivity, followed by progressive insulin resistance in the second and third trimesters driven by diabetogenic placental factors; glucose testing is therefore scheduled when insulin resistance approaches its peak and hyperglycaemia becomes detectable (11). The cost of this logic, however, is a late diagnosis. Once GDM is confirmed, only 10–15 weeks remain in which dietary, pharmacological and monitoring interventions can act, and there is essentially no opportunity to modify the early-pregnancy environment in which placental development, fetal programming and the trajectory of maternal metabolic adaptation are set.

Recognition of this limitation has brought early-onset GDM (eGDM, diagnosed before 20 weeks) into focus. The TOBOGM trial demonstrated that treatment of eGDM confers measurable, albeit modest, neonatal benefits (15). Nevertheless, guidance remains cautious: the ADA notes that for hyperglycaemia below the threshold of overt diabetes detected in early pregnancy, the benefits of treatment remain uncertain and professional society guidance is conflicting (11). Part of the uncertainty is biological — eGDM and late-onset GDM (L-GDM) may not be the same disease. In a Finnish nested case–control study, the early-pregnancy metabolome differed between eGDM and L-GDM and was modified by maternal overweight (24), and lipidomic profiling of the TOBOGM cohort identified a distinct lipid signature of eGDM, with elevated diacylglycerols and fatty acids but lower lysophosphatidylcholines and hexosylceramides (18).

Two unresolved problems therefore converge: the glycaemic thresholds for diagnosis remain contested, and the gestational timing of diagnosis is physiologically late. Both argue for tools that can identify women at high risk in the first trimester, before the diagnostic window opens — which is precisely where biomarker research has concentrated its efforts.

## 3 Pathophysiology: the metabolic soil in which biomarkers grow

Normal pregnancy is itself a state of profound metabolic adaptation. Insulin sensitivity is enhanced in early gestation, promoting maternal fat storage; thereafter, a surge of local and placental hormones — including oestrogen, progesterone, cortisol, leptin, placental lactogen and placental growth hormone — drives progressive insulin resistance in the second and third trimesters (14). To meet the fasting energy demands of pregnancy, basal endogenous (primarily hepatic) glucose production rises by approximately 30% by late gestation, despite substantially increased fasting insulin concentrations (3). Euglycaemia is preserved as long as pancreatic β-cells can mount a compensatory increase in insulin secretion.

GDM emerges when this compensation fails — in most cases, β-cell dysfunction superimposed on a background of chronic insulin resistance that antedates pregnancy (3, 14). Genetic evidence supports this formulation: most GDM-associated variants (e.g., in TCF7L2, GCK and KCNJ11) regulate insulin secretion rather than insulin action (3). The β-cell defect is amplified by several interacting disturbances: an altered adipokine profile (reduced adiponectin, which normally facilitates insulin signalling via AMP-activated protein kinase, and increased leptin); low-grade inflammation, whereby tumour necrosis factor-α and interleukin-6 impair insulin signalling through the IκB kinase/protein kinase C pathway; and lipotoxicity from elevated free fatty acids (14). More recently, the gut microbiome has been implicated as a further modifier of maternal insulin resistance, through effects on energy harvest, short-chain fatty acid production and systemic inflammation (14). Each of these disturbances leaves a measurable footprint in the maternal circulation — in lipids, fatty acids, amino acids, bile acids and microbiota-derived metabolites — and many are detectable from the first trimester, well before glucose homeostasis visibly fails (**Figure 1**). A 2015 meta-analysis further suggests that lifestyle intervention initiated before 15 weeks of gestation can reduce GDM risk (3), underscoring the therapeutic value of an early window.

**Figure 1:**
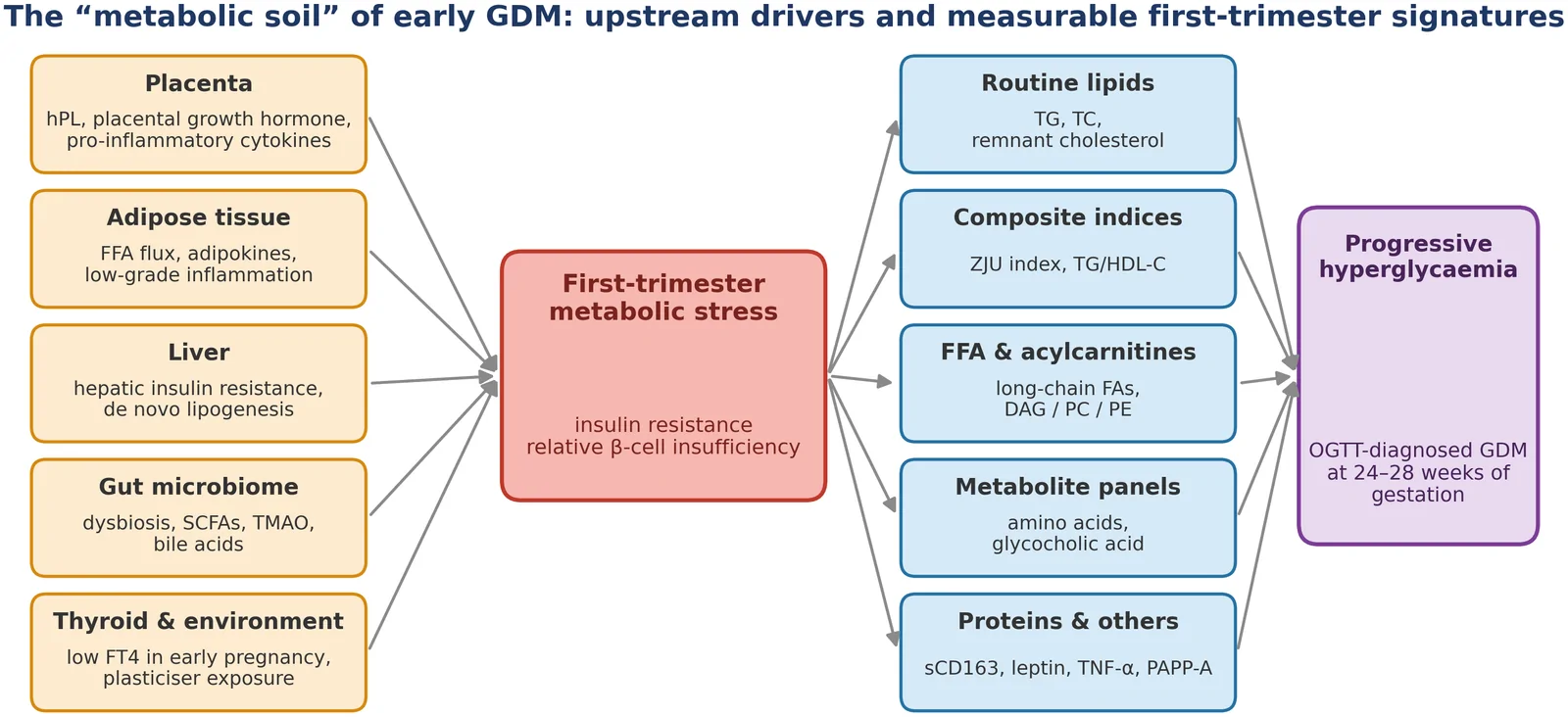
The metabolic origins of early gestational diabetes mellitus and their measurable first-trimester signatures. Placental, adipose, hepatic, gut-microbial, thyroidal and environmental contributors converge on maternal insulin resistance and relative β-cell insufficiency in early pregnancy. This metabolic stress is reflected in routine lipids, composite indices, free fatty acids and acylcarnitines, metabolite panels and circulating proteins that can be measured in the first trimester, weeks to months before the conventional oral glucose tolerance test at 24–28 weeks.

Importantly, GDM is a heterogeneous disorder. Some women exhibit predominant insulin resistance with preserved β-cell reserve (typically those with obesity), whereas others show primary β-cell failure with little insulin resistance (often lean women) (3, 24). This heterogeneity has two consequences for early prediction: no single biomarker is likely to perform uniformly across subtypes, and multi-analyte or multi-omics signatures — which capture several pathways simultaneously — are biologically better suited to the task. With this framework in mind, the following sections examine the evidence for each class of early-pregnancy biomarker.

## 4 Conventional lipid indicators and composite indices

Physiological hyperlipidaemia of pregnancy — a progressive rise in triglycerides and cholesterol that supports fetal growth — is exaggerated in women who develop GDM (14). Because lipid profiles are inexpensive, standardised and already measured in many antenatal care pathways, they have been the most extensively studied class of first-trimester predictors (**Table 1**).

The largest evidence comes from a prospective cohort of 32,411 Chinese pregnant women, in which first-trimester triglycerides (TG), total cholesterol (TC) and remnant cholesterol (RC) were each positively associated with GDM: comparing the highest with the lowest quartile, odds ratios were 1.65 (95% CI 1.36–1.99) for TG, 1.65 (1.24–2.21) for TC and 1.40 (1.19–1.64) for RC (20). Notably, the same lipid indicators also predicted pre-eclampsia, suggesting that they index a shared maternal metabolic vulnerability rather than a GDM-specific process (20). Consistent findings were reported in a Guangxi cohort, where first-trimester TG, TC and body mass index (BMI) were associated with GDM risk; a Mendelian randomisation analysis supported a causal contribution of lipid traits, and a model combining these routine variables achieved an AUC of 0.772 in the training set and 0.754 on internal validation (21).

Composite indices attempt to capture this metabolic milieu more completely. The ZJU index — integrating fasting plasma glucose, lipids, liver enzymes and BMI — predicted GDM with an AUC of 0.802 in a prospective cohort of 663 women, and was simultaneously associated with non-alcoholic fatty liver disease in pregnancy (22), echoing the growing recognition of hepatic steatosis as a companion of GDM (see Section 6). The lipid–GDM relationship is also bidirectional across gestation: in 587 women already diagnosed with GDM, a clinical model incorporating first-trimester lipids and OGTT values predicted late-pregnancy hypertriglyceridaemia with an AUC of 0.816 (95% CI 0.754–0.878) (23), a complication that itself worsens maternal and neonatal outcomes.

Taken together, conventional lipids offer moderate, reproducible predictive power at minimal cost, and composite indices perform better than any single measurement. Their limitations are equally clear: effect sizes are modest (ORs generally 1.4–1.7 per quartile), associations are confounded by BMI, diet and gestational age at sampling, and specificity is poor — the same signals predict pre-eclampsia and fatty liver. These constraints motivate the search for molecular signatures that report more directly on the underlying pathophysiology.

## 5 Free fatty acid profiles

Free fatty acids (FFAs) occupy a mechanistic junction between lipid metabolism and insulin resistance, and their qualitative composition matters as much as their quantity (27). Saturated fatty acids — notably palmitate — serve as substrates for *de novo* ceramide synthesis, and ceramide accumulation interferes with insulin signalling, providing a molecular basis for lipotoxicity. ω-6 polyunsaturated fatty acids (PUFAs) such as arachidonic and linoleic acid give rise to pro-inflammatory eicosanoids implicated in GDM pathogenesis, whereas ω-3 PUFAs (eicosapentaenoic acid and docosahexaenoic acid) are largely anti-inflammatory, making the ω-6/ω-3 balance a determinant of the inflammatory tone of pregnancy (27).

In women with GDM, circulating FFA profiles are shifted towards pro-diabetogenic patterns, and several individual FFAs measured in early pregnancy have shown potential to predict subsequent GDM (27). Beyond prediction, maternal FFAs cross and influence the placenta: altered FFA supply is associated with fetal overgrowth and macrosomia, linking maternal dyslipidaemia directly to the hallmark neonatal complication of GDM (27). Interpretation is complicated, however, by the strong influence of recent dietary intake on FFA concentrations and by inter-individual variability; FFA measurement has therefore been progressively absorbed into broader, standardised metabolomic and lipidomic platforms, to which we now turn.

## 6 Metabolomics and lipidomics: from signatures to models

Untargeted metabolomics and lipidomics profile hundreds to thousands of small molecules simultaneously and are therefore well suited to a heterogeneous disorder such as GDM. An early synthesis of the field found that reported biomarkers clustered in coherent pathways — amino acid metabolism, steroid hormone biosynthesis, glycerophospholipid metabolism and fatty acid metabolism — but that most studies were small and results were inconsistent (28). The past two years have brought larger, better-phenotyped cohorts and more rigorous analytics (**Table 2**).

In the Finnish Early Diagnosis of Diabetes in Pregnancy cohort (210 GDM, 209 controls), maternal glycaemia measured at the 12–16-week OGTT visit correlated most clearly with long-chain fatty acids and medium- and long-chain acylcarnitines, while HOMA-IR tracked tyrosine and selected phospholipids; importantly, metabolomic differences between early- and late-onset GDM were evident mainly in women with overweight (24). In the Indian STRiDE study, 49 early-pregnancy metabolites — predominantly phosphatidylcholines, sphingomyelins and triacylglycerols — distinguished women who later developed GDM, and a random-forest panel of eight metabolites achieved an AUC of 0.880 (95% CI 0.809–0.951) (16); a more conservative bootstrap mean AUC of 0.83 (95% CI 0.77–0.89) across 1,000 resamples, and single-split test estimates of up to 0.98, illustrate the optimism bias inherent in small derivation cohorts. Lipidomic profiling of 180 at-risk women from the TOBOGM trial quantified 543 lipid species across 18 classes and revealed a characteristic signature of early GDM: elevated diacylglycerols, fatty acids and ethanolamine-containing phospholipids, with lower lysophosphatidylcholines, ether-linked phosphatidylcholines and hexosylceramides (18); because lipids were profiled at the diagnostic OGTT visit (89 eGDM vs 91 controls), this constitutes a concurrent molecular signature rather than a validated model predicting later GDM.

Do these signatures improve prediction? In an ASPRE-derived dataset of 20 GDM cases and 185 controls sampled at 11 + 0–13 + 6 weeks, Tenenbaum-Gavish et al. combined first-trimester clinical and biochemical biomarkers into two exploratory derivation models: one including BMI (with insulin, sCD163 and TNF-α), intended for obese women, with an AUC of 0.95 (95% CI 0.88–0.99) and an 89% detection rate at a 10% false-positive rate; and one excluding BMI (sCD163, insulin, TNF-α, PP13 and PAPP-A), intended for non-obese women, with an AUC of 0.94 (95% CI 0.85–0.99) and an 83% detection rate at 10% FPR. These estimates derive from a single very small case series (20 GDM cases) without any internal or external validation, and the authors themselves conclude that validation in a larger independent cohort is required before clinical application.

Most instructively, in a Korean cohort (17), adding selected metabolites to established clinical factors raised the AUC from 0.631 (95% CI 0.566–0.693; clinical factors alone) to 0.849 (95% CI 0.797–0.892; clinical factors plus metabolites; p < 0.001) — direct evidence of the incremental value of omics over routine risk factors. The same study reported an exploratory mediation analysis suggesting that the associations of glycocholic acid, butanoyl carnitine and uric acid with GDM were partly accounted for by concurrently measured hepatic steatosis (estimated mediated proportions 9.7–31.9% across metabolites); as the mediator and exposure were measured concurrently, this analysis cannot establish temporality or exclude unmeasured confounding.

Across studies, several signals recur — phospholipid remodelling, glycerolipid accumulation, fatty acid and acylcarnitine excess, branched-chain and aromatic amino acids, and bile acids — and all map onto the insulin resistance, lipotoxicity and hepatic pathways outlined in Section 3. Yet no unified panel has emerged. Heterogeneity in analytical platforms, gestational windows, ethnicity, GDM subtype and adjustment for adiposity remains substantial: a 2025 scoping review counted 145 candidate first-trimester biomarkers across 133 studies, with inconsistent findings even for the most-studied proteins (19). The field thus has pathway-level convergence but marker-level divergence — a pattern typical of a research area approaching, but not yet reaching, clinical maturity.

Three observations sharpen this appraisal. First, replication is uneven across evidence classes: the glycerolipid–phospholipid axis (elevated diacylglycerols and triacylglycerols with depleted lysophosphatidylcholines) has now been detected independently in Finnish (24), Indian (16) and Australian-led (18) cohorts on different analytical platforms, and constitutes the most robust signal in the field; by contrast, most amino-acid and bile-acid findings rest on single cohorts. Second, statistical practice varies widely — only a minority of studies apply false-discovery-rate control, and fewer still adjust for maternal adiposity, the strongest shared confounder of metabolite levels and GDM risk (19, 24). Third, several of the highest reported AUCs were estimated in the same small samples used for feature selection, so optimism bias is likely; the 0.88–0.95 range should be read as an upper bound until external validation demonstrates otherwise (16, 25).

## 7 Gut microbiome and microbiota-derived metabolites

The gut microbiome influences host insulin sensitivity through energy harvest, short-chain fatty acid production and low-grade inflammation (14), and pregnancy itself remodels maternal microbial communities. The most comprehensive analysis to date integrated 2,717 mothers and infants from nine global cohorts: network modelling identified a core gut microbial guild (XNP_Guild1) that remained stable and functionally cohesive across healthy, transitional (“grey zone”) and GDM states, yet was significantly associated with disease progression and with risk already in early pregnancy; exploratory intergenerational analyses further suggested vertical transmission of this guild with potential effects on neonatal growth (29). The concept of a microbial “grey zone” — an ecological transition state between health and GDM — is attractive for prediction, because it implies that microbiome features may shift before clinical hyperglycaemia appears.

Microbiota-derived metabolites provide a more directly measurable readout. The trimethylamine N-oxide (TMAO) pathway links microbial metabolism of dietary choline and L-carnitine to host glucose and lipid homeostasis. In a nested case–control study with serial sampling, women with GDM showed progressive decreases in L-carnitine alongside rising choline and trimethylamine across gestation; first-trimester L-carnitine and trimethylamine were positively associated with GDM, and mixture analyses (BKMR, WQS, quantile g-computation) indicated that the joint effect of TMAO-related metabolites was strongest in the third trimester and driven mainly by trimethylamine (30). The trimester-dependent, non-linear associations caution against single-timepoint interpretations and argue for longitudinal designs.

The appeal of microbiome-based prediction lies in its modifiability — diet, prebiotics and probiotics are potential levers — but the field faces pronounced geographic and dietary variability in baseline microbiota, and causality remains unresolved. At present, microbial features are best viewed as candidate components of multi-omics panels rather than standalone predictors.

## 8 Other emerging contributors: thyroid function and environmental exposures

Two further lines of evidence widen the aetiological lens. First, thyroid function: in a prospective bi-centre cohort of 1,486 women, the first-trimester FT4/TSH ratio was significantly higher in women who developed GDM (incidence 23.0%), with the association most pronounced in women carrying a male fetus (31); conversely, in a Belgian cohort, lower first-trimester FT4 was associated with subsequent need for insulin therapy among women with GDM (32). These complementary findings point to a bidirectional thyroid–glucose interaction, and the fetal-sex specificity hints at endocrine heterogeneity that most prediction models ignore. Second, environmental exposures: in a nested case–control study (286 pairs) within a Wuhan birth cohort, gestational exposure to several non-phthalate plasticisers (e.g., dimethyl azelate) was associated with elevated GDM risk, with a joint mixture effect and partial mediation through the plasma lipidome (33). Both domains are nascent, but they share an important property: they are modifiable or avoidable, and they demonstrate that GDM risk signals extend well beyond the glucose-centred panel — reinforcing the case for broad, hypothesis-free profiling.

## 9 From biomarkers to models: closing the translational gap

### 9.1 Comparative model performance

When prediction models are arrayed by input type, a consistent gradient emerges (**Table 3**). Models built on clinical risk factors alone perform modestly (AUCs 0.63–0.69); adding routine lipids and composite indices raises performance to approximately 0.75–0.82, and — importantly — at least one such model has now undergone genuine external validation (AUC 0.759 in an independent hospital cohort of 390 women). Omics-enhanced models report higher derivation-stage AUCs (0.83–0.95), and two within-study comparisons provide the most direct evidence of incremental value: clinical factors alone versus clinical factors plus metabolites (AUC 0.631 [0.566–0.693] versus 0.849 [0.797–0.892]) and conventional risk factors alone versus conventional factors plus an eight-metabolite panel (0.655 versus 0.881). Two cautions are essential. First, the two highest estimates in the literature (AUCs of 0.94–0.95) derive from a single dataset of only 20 GDM cases and have never been validated; second, where resampling-based estimates are reported, they are materially lower than headline test-set estimates (bootstrap mean 0.83 versus 0.880). The apparent performance gradient should therefore be read as hypothesis-generating rather than as established evidence of clinical superiority.

### 9.2 Methodological gaps

These encouraging numbers must be weighed against four caveats. First, the best-performing models derive from the smallest samples: the panels reporting AUCs of 0.88–0.95 were trained on 100–205 pregnancies (16, 25), whereas models using routine variables involved thousands (20). High-dimensional omics data in small derivation cohorts are exquisitely prone to overfitting, and machine-learning approaches (random forest, LASSO) amplify this risk when internal validation is the only safeguard (16, 23). Second, external validation is rare; most models have never been tested outside their derivation cohort, let alone across platforms or ethnicities (19). Third, reporting is inconsistent — a problem the TRIPOD statement was designed to prevent (34) — and formal risk-of-bias assessment with tools such as PROBAST would likely rate much of this literature at high or unclear risk (35). Fourth, analytical heterogeneity (LC-MS platforms, relative versus absolute quantification, gestational windows ranging from 8 to 20 weeks) makes cross-study pooling premature (19).

### 9.3 Beyond discrimination: calibration and clinical utility

Nearly all models in this literature are evaluated on discrimination alone, yet two further properties determine clinical value. Calibration — whether predicted risks match observed event rates — is rarely reported, and machine-learning models are frequently miscalibrated out of the box; an uncalibrated model with an AUC of 0.90 can mislead more than a calibrated model at 0.75. Clinical utility is the second gap: no study has yet applied decision-curve analysis to show that biomarker-guided management yields greater net benefit than universal OGTT screening or clinical factors alone. Simple arithmetic illustrates the stakes: at a GDM prevalence of 15%, a model with 80% sensitivity and 80% specificity yields a positive predictive value of only about 41%, meaning that most biomarker-positive women would be false alarms subjected to intensified surveillance. Until models are calibrated, externally validated and shown to improve net benefit at plausible risk thresholds, high AUCs remain a necessary but far from sufficient condition for implementation.

### 9.4 From prediction to prevention

Even a perfectly validated model is clinically inert without an intervention pathway. The logic of early prediction rests on two assumptions: that risk can be identified before 20 weeks, and that acting on that risk improves outcomes. The first is increasingly plausible; the second has only partial support — TOBOGM showed modest neonatal benefit from treating early-diagnosed GDM (15), and professional guidance on managing early-pregnancy hyperglycaemia remains conflicting (11). What is missing is the closed loop: trials that randomise women stratified by biomarker-based risk to early lifestyle or pharmacological intervention versus standard care, with perinatal and long-term outcomes.

### 9.5 A pragmatic roadmap

Progress requires (i) large, multi-ethnic prospective cohorts with first-trimester sampling and standardised gestational windows; (ii) absolute quantification on harmonised platforms to enable transferable thresholds; (iii) head-to-head comparison and external validation of clinical, omics and combined models, reported per TRIPOD (34); (iv) evaluation of simplified, low-cost panels (3–8 analytes) that could be implemented in routine laboratories, as exemplified by the eight-metabolite STRiDE panel (16); and (v) cost-effectiveness and implementation studies, particularly in the high-burden, resource-diverse settings of Asia where GDM prevalence and early-onset diabetes risk are greatest (4, 16) (**Figure 2**).

**Figure 2:**
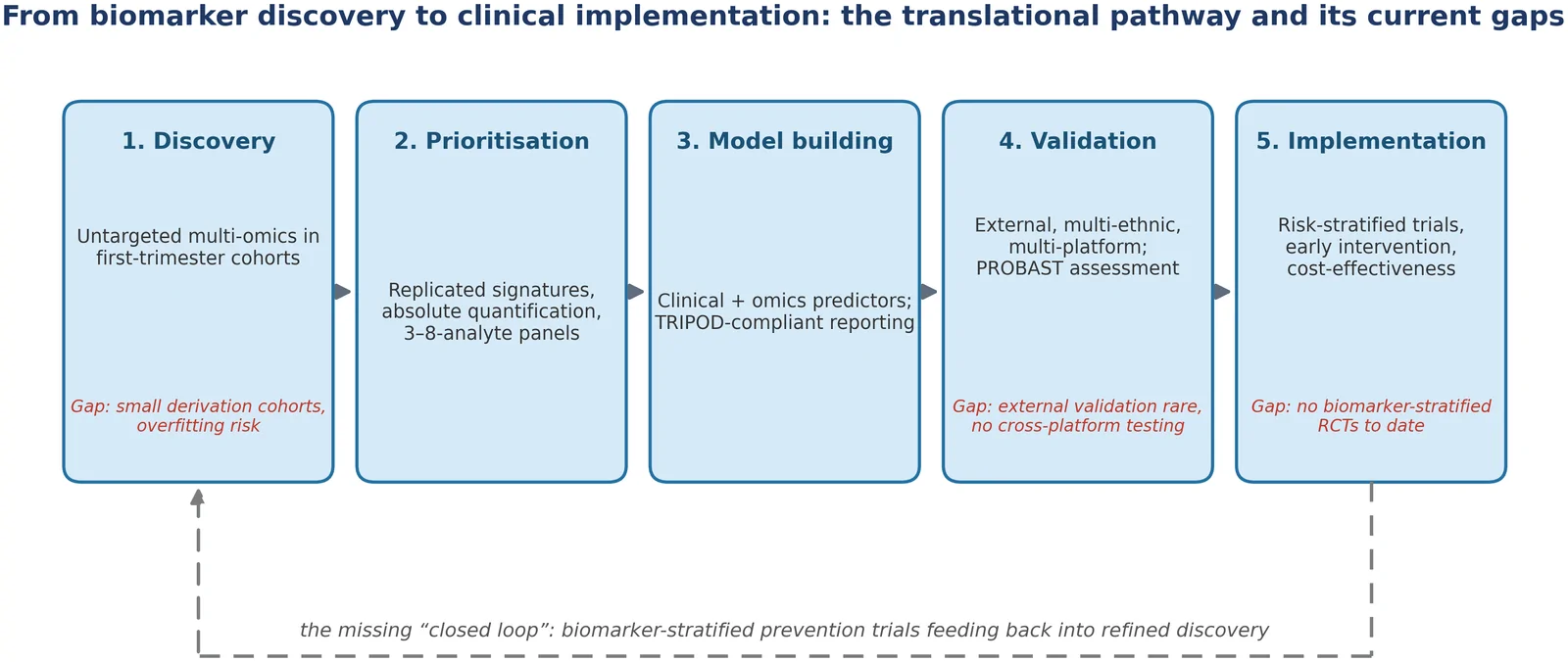
The translational pathway from biomarker discovery to clinical implementation for early prediction of gestational diabetes mellitus, and its current gaps. Progress requires untargeted discovery in well-phenotyped first-trimester cohorts; prioritisation of replicated, absolutely quantified signatures into parsimonious panels; TRIPOD-compliant model development; external, multi-ethnic and multi-platform validation with formal risk-of-bias assessment; and, ultimately, biomarker-stratified prevention trials. Red markers indicate the principal gaps identified in this review: overfitting in small derivation cohorts, rare external validation, and the absence of biomarker-stratified randomised trials. The dashed loop depicts the feedback from prevention trials to refined discovery that is currently missing.

## 10 Treatment and the long arc of maternal and offspring health

Once GDM is diagnosed, lifestyle intervention is the foundation of management, with insulin the preferred pharmacotherapy when treatment is required; oral agents cross the placenta and remain second-line (36, 37). From the standpoint of early prediction, however, the clinically decisive question is narrower: which interventions could realistically follow first-trimester risk stratification, and what evidence supports them? Lifestyle intervention initiated before 15 weeks may reduce GDM risk, and TOBOGM showed modest neonatal benefit from treating GDM diagnosed before 20 weeks — but neither trial enriched for biomarker-defined risk, and GEMS showed that lowering diagnostic thresholds alone does not improve outcomes. What is missing is therefore the closed loop: randomised trials that stratify women by validated first-trimester biomarker panels and randomise high-risk women to early targeted intervention versus standard care, with perinatal and long-term metabolic endpoints.

The stakes of GDM extend decades beyond delivery: affected women carry an approximately seven-fold relative risk of type 2 diabetes, with the absolute burden concentrated in the early postpartum years, and they face elevated risks of cardiovascular and cerebrovascular disease; their offspring show higher rates of childhood adiposity and impaired glucose metabolism. GDM is thus best understood as a metabolic stress test that unmasks a lifelong trajectory — and an opportunity for prevention. Women identified as high-risk by first-trimester biomarkers are precisely the population in whom intensive postpartum surveillance and diabetes-prevention programmes may yield the greatest return.

## 11 Conclusions and future directions

GDM is diagnosed too late: the 24–28-week OGTT opens a window of barely one trimester in which to act, while the metabolic disturbances that define the disease are already measurable in the first trimester. Multi-omics studies have now mapped these disturbances with increasing resolution — conventional lipids and composite indices offer moderate, low-cost prediction; metabolomic and lipidomic signatures add substantial incremental value over clinical factors alone; and gut-microbiome features, thyroid function and environmental exposures are widening the aetiological frame. The recurring signals — phospholipid remodelling, glycerolipid accumulation, fatty acid and acylcarnitine excess, branched-chain amino acids and bile acids — converge on insulin resistance, lipotoxicity and hepatic steatosis.

The challenge ahead is no longer discovery but translation. The field must move from small, single-platform derivation studies to large, harmonised, multi-ethnic cohorts; from relative to absolute quantification; from internal to external validation under TRIPOD and PROBAST standards; and, ultimately, to trials that test whether biomarker-guided early intervention improves outcomes that matter to mothers and children. Given the disproportionate and rising burden of GDM in Asian populations, and their earlier onset of metabolic disease at lower BMI, these populations should be at the centre — not the periphery — of this translational effort. If these steps are taken, first-trimester prediction could shift GDM care from late diagnosis and damage control towards genuine prevention.

## References

[1] World Health Organization . Diagnostic criteria and classification of hyperglycaemia first detected in pregnancy. (WHO/NMH/MND/13.2). Geneva: World Health Organization (2013). 24199271

[2] Zhu Y Zhang C . Prevalence of gestational diabetes and risk of progression to type 2 diabetes: a global perspective. Curr Diabetes Rep. (2016) 16:7. doi: 10.1007/s11892-015-0699-x 26742932 PMC6675405

[3] McIntyre HD Catalano P Zhang C Desoye G Mathiesen ER Damm P . Gestational diabetes mellitus. Nat Rev Dis Primers. (2019) 5:47. doi: 10.1038/s41572-019-0098-8 31296866

[4] Gao C Sun X Lu L Liu F Yuan J . Prevalence of gestational diabetes mellitus in mainland China: a systematic review and meta-analysis. J Diabetes Investig. (2019) 10:154–62. doi: 10.1111/jdi.13086 PMC6319492 29683557

[5] HAPO Study Cooperative Research Group Metzger BE Lowe LP Dyer AR Trimble ER Chaovarindr U . Hyperglycemia and adverse pregnancy outcomes. N Engl J Med. (2008) 358:1991–2002. doi: 10.1056/NEJMoa0707943 18463375

[6] Bellamy L Casas JP Hingorani AD Williams D . Type 2 diabetes mellitus after gestational diabetes: a systematic review and meta-analysis. Lancet. (2009) 373:1773–9. doi: 10.1016/s0140-6736(09)60731-5 19465232

[7] Dennison RA Chen ES Green ME Legard C Kotecha D Farmer G . The absolute and relative risk of type 2 diabetes after gestational diabetes: a systematic review and meta-analysis of 129 studies. Diabetes Res Clin Pract. (2021) 171:108625. doi: 10.1016/j.diabres.2020.108625 33333204 PMC7610694

[8] Xie W Wang Y Xiao S Qiu L Yu Y Zhang Z . Association of gestational diabetes mellitus with overall and type specific cardiovascular and cerebrovascular diseases: systematic review and meta-analysis. BMJ. (2022) 378:e070244. doi: 10.1136/bmj-2022-070244 36130740 PMC9490552

[9] Lowe WL Jr Scholtens DM Lowe LP Kuang A Nodzenski M Talbot O . Association of gestational diabetes with maternal disorders of glucose metabolism and childhood adiposity. JAMA. (2018) 320:1005–16. doi: 10.1001/jama.2018.11628 30208453 PMC6143108

[10] International Association of Diabetes and Pregnancy Study Groups Consensus Panel Metzger BE Gabbe SG Persson B Buchanan TA Catalano PA . International association of diabetes and pregnancy study groups recommendations on the diagnosis and classification of hyperglycemia in pregnancy. Diabetes Care. (2010) 33:676–82. doi: 10.2337/dc10-0719 PMC2827530 20190296

[11] American Diabetes Association Professional Practice Committee . 15. Management of diabetes in pregnancy: Standards of Care in Diabetes—2026. Diabetes Care. (2026) 49:S321–38. doi: 10.2337/dc26-S015 PMC12690181 41358885

[12] Crowther CA Hiller JE Moss JR McPhee AJ Jeffries WS Robinson JS . Effect of treatment of gestational diabetes mellitus on pregnancy outcomes. N Engl J Med. (2005) 352:2477–86. doi: 10.1056/nejmoa042973 15951574

[13] Landon MB Spong CY Thom E Carpenter MW Ramin SM Casey B . A multicenter, randomized trial of treatment for mild gestational diabetes. N Engl J Med. (2009) 361:1339–48. doi: 10.1056/nejmoa0902430 19797280 PMC2804874

[14] Plows JF Stanley JL Baker PN Reynolds CM Vickers MH . The pathophysiology of gestational diabetes mellitus. Int J Mol Sci. (2018) 19:3342. doi: 10.3390/ijms19113342 30373146 PMC6274679

[15] Simmons D Immanuel J Hague WM Teede H Nolan CJ Peek MJ . Treatment of gestational diabetes mellitus diagnosed early in pregnancy. N Engl J Med. (2023) 388:2132–44. doi: 10.1056/nejmoa2214956 37144983

[16] Sharma S Subrahmanyam YV Gupta P Vadivel S Deepa M Tandon A . Precision integrated identification of predictive first-trimester metabolomics signatures for early detection of gestational diabetes mellitus. Cardiovasc Diabetol. (2025) 24:434. doi: 10.1186/s12933-025-02978-0 41239402 PMC12619188

[17] Jung Y Lee SM Lee J Kim Y Lee W Koo JN . Metabolomic profiling reveals early biomarkers of gestational diabetes mellitus and associated hepatic steatosis. Cardiovasc Diabetol. (2025) 24:125. doi: 10.1186/s12933-025-02645-4 40114104 PMC11927189

[18] Saadati S Godini R Jona E Barlow CK Neven AC Nolan CJ . Lipidomic profiling reveals a distinct lipidomic signature of early gestational diabetes. Commun Med (Lond). (2026). doi: 10.1038/s43856-026-01765-6 42401700 PMC13714274

[19] Swinburne M Krasner S Mathewlynn S Collins S . First-trimester biomarkers of gestational diabetes mellitus: a scoping review. Acta Obstet Gynecol Scand. (2025) 104:1838–48. doi: 10.1111/aogs.70046 40879308 PMC12451201

[20] Tang J Chen Y Sun M Luo M Chen Y Chen K . The association between early pregnancy maternal lipid indicators with gestational diabetes mellitus and pre-eclampsia. J Lipid Res. (2025) 66:100942. doi: 10.1016/j.jlr.2025.100942 41232673 PMC12732318

[21] Wang Y He X Nie L Yue C Li R Mo L . Association between blood lipids, BMI and gestational diabetes mellitus in early pregnancy: a clinical study and Mendelian randomization. Front Endocrinol (Lausanne). (2026) 17:1870557. doi: 10.3389/fendo.2026.1870557 42630205 PMC13493276

[22] Xu Z Li X Wang H Xu L Li C . ZJU index as a predictive biomarker of gestational diabetes mellitus: a prospective cohort analysis. Front Nutr. (2025) 12:1570771. doi: 10.3389/fnut.2025.1570771 40584102 PMC12202635

[23] Zhao Y Zhang J Jin X . Early prediction of late-pregnancy hypertriglyceridemia in women with gestational diabetes: development and internal validation of a clinical risk model. Front Endocrinol (Lausanne). (2026) 17:1845573. doi: 10.3389/fendo.2026.1845573 42434302 PMC13350170

[24] Masalin S Klåvus A Rönö K Koistinen HA Koistinen V Kärkäinen O . Analysis of early-pregnancy metabolome in early- and late-onset gestational diabetes reveals distinct associations with maternal overweight. Diabetologia. (2024) 67:2539–54. doi: 10.1007/s00125-024-06237-x 39083240 PMC11519293

[25] Tenenbaum-Gavish K Sharabi-Nov A Binyamin D Møller HJ Danon D Rothman L . First trimester biomarkers for prediction of gestational diabetes mellitus. Placenta. (2020) 101:80–9. doi: 10.1016/j.placenta.2020.08.020 32937245

[26] Crowther CA Samuel D McCowan LME Edlin R Tran T McKinlay CJ . Lower versus higher glycemic criteria for diagnosis of gestational diabetes. N Engl J Med. (2022) 387:587–98. doi: 10.1056/NEJMoa2204091 36070709

[27] Du H Li D Molive LM Wu N . Advances in free fatty acid profiles in gestational diabetes mellitus. J Transl Med. (2024) 22:180. doi: 10.1186/s12967-024-04922-4 38374136 PMC10875910

[28] Chen Q Francis E Hu G Chen L . Metabolomic profiling of women with gestational diabetes mellitus and their offspring: review of metabolomics studies. J Diabetes Complications. (2018) 32:512–23. doi: 10.1016/j.jdiacomp.2018.01.007 29506818

[29] Du H Lin Q He X Yang B Huang Y Li Q . Dynamic involvement of the core gut microbiome XNP_Guild1 in the evolution of gestational diabetes mellitus. Gut Microbes. (2026) 18:2623353. doi: 10.1080/19490976.2026.2623353 41620634 PMC12867416

[30] Wu Y Ni Z Liu S Fang S Li Y Lu Q . Longitudinal metabolomics reveals dynamic associations of trimethylamine N-oxide and precursors with the progression of gestational diabetes. BMC Pregnancy Childbirth. (2026) 26:612. doi: 10.1186/s12884-026-09088-z 42021207 PMC13235132

[31] Song S Zhang Y Qiao X Duo Y Xu J Zhang J . Thyroid FT4-to-TSH ratio in the first trimester is associated with gestational diabetes mellitus in women carrying male fetus: a prospective bi-center cohort study. Front Endocrinol (Lausanne). (2024) 15:1427925. doi: 10.3389/fendo.2024.1427925 39678197 PMC11637856

[32] Jelloul E Lomré M Kleynen P Sitoris G Grabczan L Veltri F . Low FT4 levels in early pregnancy are associated with higher insulin therapy need in women with gestational diabetes mellitus. Eur Thyroid J. (2026) 15:ETJ250206. doi: 10.1530/ETJ-25-0206 PMC13138595 41439610

[33] Mao Y Tang S Liu H Huang Q Chen D Li Y . Association of non-phthalate plasticizer exposure during early pregnancy with gestational diabetes mellitus: the potential mediation effect of plasma lipidome. Ecotoxicol Environ Saf. (2025) 299:118405. doi: 10.1016/j.ecoenv.2025.118405 40424727

[34] Collins GS Reitsma JB Altman DG Moons KGM TRIPOD Group . Transparent reporting of a multivariable prediction model for individual prognosis or diagnosis (TRIPOD): the TRIPOD statement. Ann Intern Med. (2015) 162:55–63. doi: 10.1186/s12916-014-0241-z 25560714

[35] Wolff RF Moons KGM Riley RD Whiting PF Westwood M Collins GS . PROBAST: a tool to assess the risk of bias and applicability of prediction model studies. Ann Intern Med. (2019) 170:51–8. doi: 10.7326/m18-1376 30596875

[36] Rowan JA Hague WM Gao W Battin MR Moore MP MiG Trial Investigators . Metformin versus insulin for the treatment of gestational diabetes. N Engl J Med. (2008) 358:2003–15. doi: 10.1056/nejmoa0707193 18463376

[37] Langer O Conway DL Berkus MD Xenakis EM Gonzales O . A comparison of glyburide and insulin in women with gestational diabetes mellitus. N Engl J Med. (2000) 343:1134–8. doi: 10.1056/nejm200010193431601 11036118

